# Combined use of preoperative lymphocyte counts and the post/preoperative lymphocyte count ratio as a prognostic marker of recurrence after curative resection of stage II colon cancer

**DOI:** 10.18632/oncotarget.23510

**Published:** 2017-12-20

**Authors:** Seiichi Shinji, Yoshibumi Ueda, Takeshi Yamada, Michihiro Koizumi, Yasuyuki Yokoyama, Goro Takahashi, Masahiro Hotta, Takuma Iwai, Keisuke Hara, Kohki Takeda, Mikihiro Okusa, Hayato Kan, Eiji Uchida

**Affiliations:** ^1^ Department of Gastrointestinal and Hepato-Biliary-Pancreatic Surgery, Nippon Medical School, Tokyo, Japan; ^2^ Graduate School of Arts and Sciences, The University of Tokyo, Tokyo, Japan; ^3^ AMED-PRIME, Japan Agency for Medical Research and Development, Tokyo, Japan

**Keywords:** lymphocyte count, post/preoperative lymphocyte count ratio, colon cancer, prognostic marker, stage II

## Abstract

**Purpose:**

Diagnostic markers for recurrence of colorectal cancer have not been established. The aim of the present study was to identify new diagnostic markers for recurrence after curative surgery of stage II colon cancer.

**Materials and Methods:**

In this study, the prognostic values of the preoperative lymphocyte count and the post/preoperative lymphocyte count ratio (PPLR) were evaluated in 142 patients with localized colon cancer treated with surgery at a single medical center. The associations of patient demographics, blood chemistry, and serum biochemical indices with recurrence-free survival (RFS) and cancer-specific survival (CSS) were examined by univariate and multivariate analyses.

**Results:**

Receiver operating characteristic (ROC) curve analysis showed that the optimal cut-off values of the lymphocyte count and PPLR were, respectively, 1555.2/μl and 1.151 for RFS. On univariate analysis, tumor depth of invasion, carbohydrate antigen 19-9 (CA19-9), and preoperative low lymphocyte count (≤1555.2/μl) were all correlated with poorer RFS (*p* < 0.05). On multivariate analysis, T4, low lymphocyte count, and low PPLR were independent predictors of poor RFS. Furthermore, the patients were categorized into four categories based on preoperative lymphocyte count high/low and PPLR high/low. Patients with a low preoperative lymphocyte count and low PPLR had the poorest RFS and CSS compared to the other patients.

**Conclusion:**

The combination of the preoperative lymphocyte count and the PPLR appears to be a potential marker for predicting recurrence of stage II colon cancer.

## INTRODUCTION

Colorectal cancer (CRC) is the third most commonly diagnosed malignancy, the fourth leading cause of cancer-related deaths worldwide [1], and the second most common cause of cancer-related death in Japan [2]. During the past two decades, advances in chemotherapy protocols have drastically decreased the risk of cancer recurrence and improved overall survival time of patients with stages III and IV [3–8]. On the other hand, use of chemotherapy among patients with stage II colon cancer is controversial [9, 10], as is the usefulness of adjuvant chemotherapy after surgery. Therefore, better prognostic markers are needed to improve the outcomes of patients with CRC.

Currently, several predictors for recurrence of curatively resected CRCs have been proposed. For instance, carcinoembryonic antigen (CEA) is a prognostic marker for long-term CRC recurrence [11, 12]. Additionally, the hemogram of the peripheral blood could be a useful diagnostic marker. For instance, the lymphocyte count [13, 14], the systemic inflammation score (SIS) [15], the lymphocyte and monocyte ratio (LMR) [16], the neutrophil and lymphocyte ratio (NLR) [17], the platelet to lymphocyte ratio (PLR) [18], and the platelet distribution width (PDW) [17] have been reported to be correlated with disease-free survival (DFS) and overall survival (OS) in patients with CRC. However, according to the European Group on Tumor Markers (EGTM) published guidelines, CEA is more useful for postoperative surveillance than as a predictor of recurrence [19]. Other predictors have been emerging over the last several years, and they are not yet practically useful. Therefore, more potential prognostic markers are required. In the present study, the aim was to identify other prognostic markers for CRC, and it was found that combined use of the lymphocyte count and the post/preoperative lymphocyte count ratio (PPLR) was an effective prognostic marker.

## RESULTS

### Patients’ characteristics

**Figure 1:**
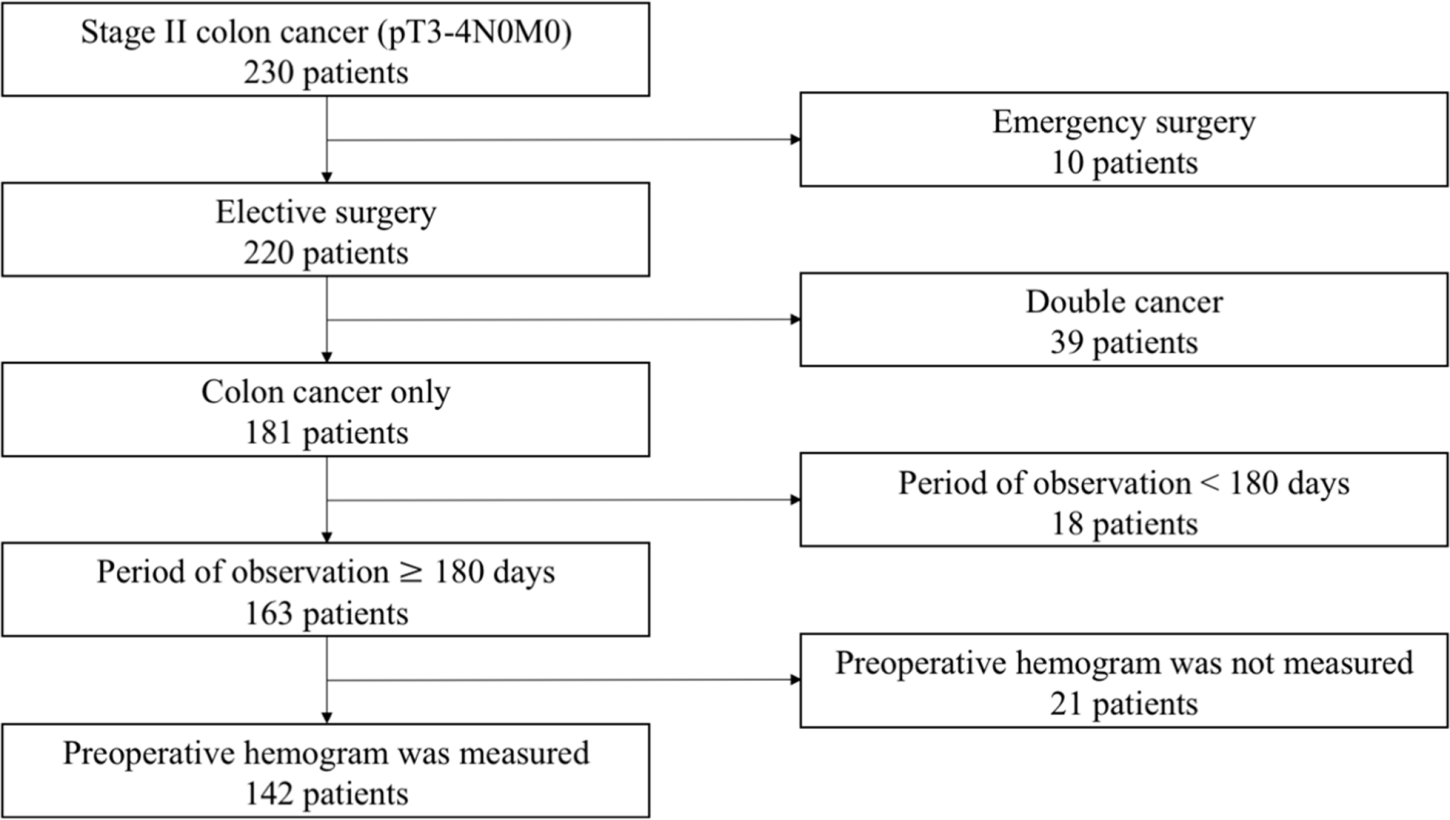
Flow chart of the 230 patients enrolled in this study

A total of 142 patients with Stage II colon cancer who underwent curative surgery in our hospital between January 2008 and December 2014 were enrolled in the study (Figure 1). The male to female ratio was around 1.3: 1. The mean age at the time of diagnosis was 72 years (range, 43 to 94 years). In the present study cohort, 17 patients (12.0%) developed tumor recurrence during the follow-up period. Among them, including overlaps, 4 patients showed local recurrence, 5 patients had peritoneal metastases, 5 patients had liver metastases, 4 patients had lung metastases, and 1 had brain metastasis. Five patients (3.5%) died from cancer recurrence. Laboratory results, including various blood cell counts, are shown in Table 1. The median follow-up duration was 47.0 months (range, 6.1 to 116.3 months).

**Table 1:** Patients’ baseline demographic and clinical characteristics

Parameter		Patients (n=142)
Age (y)	< 60	16 (11.3)
	≥ 60	126 (88.7)
Sex	F	62 (43.7)
	M	80 (56.3)
Location	Left	81 (57.0)
	Right	61 (43.0)
Differentiation	Pap	4 (2.8)
	Well	54 (38.0)
	Moderately	76 (53.5)
	Poor	1 (0.7)
	Muc	7 (4.9)
Tumor invasion depth	T3	120 (84.5)
	T4	22 (15.5)
Lymphatic involvement	Negative	37 (26.1)
	Positive	105 (73.9)
Venous involvement	Negative	39 (27.5)
	Positive	103 (72.5)
Diameter	< 5 cm	63 (45.0)
	≥ 5 cm	77 (55.0)
CEA	< 5 ng/ml	93 (69.4)
	≥ 5 ng/ml	41 (30.6)
CA19-9	< 37 U/ml	112 (85.5)
	≥ 37 U/ml	19 (14.5)
Age (y)		72.1 ± 10.8
WBC count (×10^2^/μl)		66.4 ± 21.9
Neutrophil count (×10^2^/μl)		45.0 ± 20.2
Lymphocyte count (×10^2^/μl)		15.5 ± 5.8
Monocyte count (×10^2^/μl)		4.0 ± 1.7
Platelet count (×10^4^/μl)		27.8 ± 9.7
Albumin (g/dl)		3.8 ± 0.6
Adjuvant chemotherapy	No	128 (80.1)
	Yes	14 (9.9)

### ROC curve analysis

**Figure 2:**
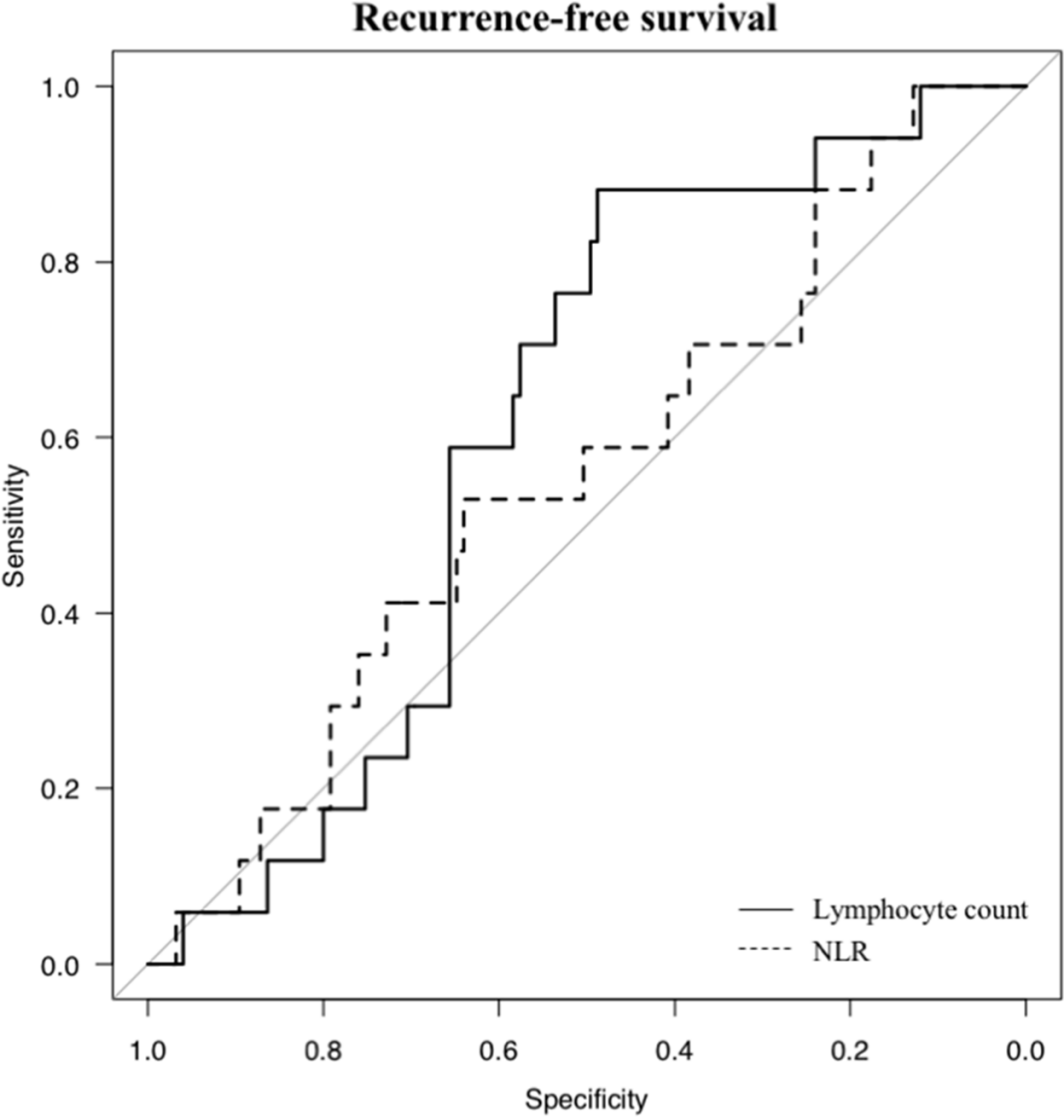
Receiver operating characteristic curve analyses For RFS, the lymphocyte count is represented by the solid line with an area under the curve (AUC) of 0.61 (95% CI, 0.495-0.728), with a sensitivity of 88.2% and a specificity of 48.8%, and NLR is represented by the dotted line with an AUC of 0.56 (95% CI, 0.411-0.698), with a sensitivity of 47.1% and specificity of 64.0%

Applying receiver operating characteristic (ROC) curve analysis, the cut-off value for the lymphocyte count for RFS was 1555.2/μl, and the area under the curve (AUC) was 0.61 (95% confidence interval (CI), 0.495-0.728), with a sensitivity of 88.2% and a specificity of 48.8%. For NLR, the optimal cut-off value for RFS was 3.197, and the AUC was 0.56 (95% CI, 0.411-0.698), with a sensitivity of 47.1% and a specificity of 64.0% (Figure 2, Table 2). These data suggest that the preoperative lymphocyte count is superior to NLR as a prognostic marker. Furthermore, for PPLR, the optimal cut-off value for RFS was 1.151, and the AUC was 0.51 (95% CI, 0.362-0.65), with a sensitivity of 64.7% and a specificity of 41.6% (Table 2).

**Table 2:** Receiver operating characteristic (ROC) curve analyses for recurrence-free survival (RFS)

	AUC	95% CI	Sensitivity	Specificity	Cut-off
Lymphocyte count	0.61	0.495-0.728	88.2%	48.8%	1555.2
NLR	0.56	0.411-0.698	47.1%	64.0%	3.197
PPLR	0.51	0.362-0.65	64.7%	41.6%	1.151

AUC: area under the curve, CI: confidence interval, NLR: neutrophil and lymphocyte ratio, PPLR: post/preoperative lymphocyte count ratio.

### Recurrence-free survival and cancer-specific survival

**Figure 3:**
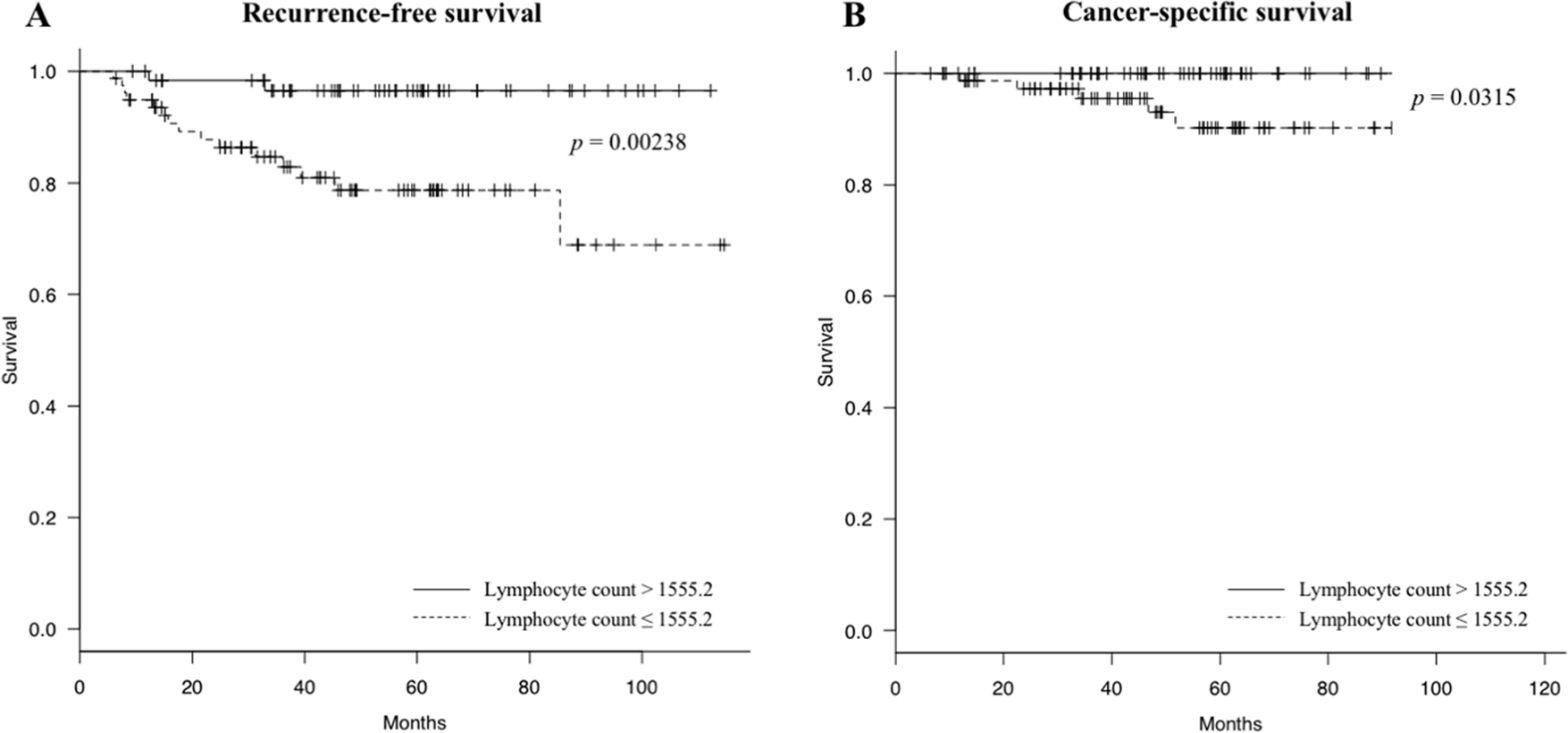
Recurrence-free survival (RFS) and cancer-specific survival (CSS) curves grouped by lymphatic cell count **(A)** Patients with a low lymphocyte count ≤1555.2/μl (dotted line) have a significantly worse RFS compared to patients with a high lymphocyte count (> 1555.2/μl) (solid line) (log-rank *p* = 0.00238); **(B)** Patients with a low lymphocyte count ≤1555.2/μl (dotted line) have a significantly worse CSS than patients with a high lymphocyte count (> 1555.2/μl) (solid line) (log-rank *p* = 0.0315).

Kaplan-Meier analysis and the log-rank test were used to evaluate differences in RFS between group pairs defined by the lymphocyte count. Patients with a low lymphocyte count (≤1555.2/μl, n = 79) had a significantly shorter RFS than those with a high lymphocyte count (>1555.2/μl, n = 63) (*p* = 0.00238) (Figure 3A). Furthermore, patients with a low lymphocyte count (≤1555.2/μl) also had a significantly worse CSS than those with a high lymphocyte count (*p* = 0.0315) (Figure 3B). Table 3 shows the distribution of the clinical background characteristics of the studied patients divided into two groups by the lymphocyte count cut-off of 1555.2/μl. Significant between-group differences were found for recurrence (*p* = 0.004), age (*p* = 0.003), lymphatic involvement (*p* = 0.036), diameter (*p* = 0.042), and WBC count (*p* = 0.005). In the group with a low lymphocyte count (≤1555.2/μl), 7 of 79 patients had adjuvant chemotherapy, while 7 of 63 patients had adjuvant chemotherapy in the group with a high lymphocyte count (>1555.2/μl); there was no between-group difference (*p* = 0.779). These data suggest that the lymphocyte count is a predictive marker for CRC recurrence.

**Table 3:** Relationships between clinical characteristics and the preoperative lymphocyte count

Parameter	Lymphocyte count (/μl)		p
	≤ 1555.2	> 1555.2	
	(n=79)	(n=63)	
Recurrence			0.004
No	64 (81)	61 (97)	
Yes	15 (19)	2 (3)	
Age (y)	74.5 ± 10.7	69.1 ± 10.3	0.003
Sex			0.130
Male	40 (51)	40 (63)	
Female	39 (49)	23 (37)	
Location			0.177
Right colon	38 (48)	23 (37)	
Left colon	41 (52)	40 (63)	
Differentiation			1.000
Well/Moderately/Pap	74 (94)	60 (95)	
Poor/Muc	5 (6)	3 (5)	
Tumor invasion depth			0.817
T3	66 (84)	54 (86)	
T4	13 (16)	9 (14)	
Lymphatic involvement			0.036
Negative	15 (19)	22 (35)	
Positive	64 (81)	41 (65)	
Venous involvement			0.573
Negative	20 (25)	19 (30)	
Positive	59 (75)	44 (70)	
Diameter			0.042
< 5 cm	29 (37)	34 (55)	
≥ 5 cm	49 (63)	28 (45)	
CEA			0.576
< 5 ng/ml	49 (67)	44 (72)	
≥ 5 ng/ml	24 (33)	17 (28)	
CA19-9			0.809
< 37 U/ml	61 (85)	51 (86)	
≥ 37 U/ml	11 (15)	8 (14)	
CEA (ng/ml)	36.9 ± 218	6.7 ± 12.7	0.282
CA19-9 (U/ml)	38.7 ± 97.7	21.2 ± 39.3	0.200
WBC count (×10^2^/μl)	61.7 ± 21.3	72.1 ± 21.5	0.005
Neutrophil count (×10^2^/μl)	45.1 ± 20.1	44.8 ± 20.4	0.949
Lymphocyte count (×10^2^/μl)	11.4 ± 2.6	20.6 ± 4.5	< 0.001
Monocyte count (×10^2^/μl)	3.7 ± 1.8	4.3 ± 1.6	0.051
Platelet count (×10^4^/μl)	26.9 ± 10.0	29.0 ± 9.3	0.216
Albumin (g/dl)	3.77 ± 0.58	3.87 ± 0.54	0.27
Adjuvant chemotherapy			0.779
Yes	7 (9)	7 (11)	
No	72 (91)	56 (89)	

### Univariate and multivariate analyses for RFS

In the group with a low lymphocyte count (≤1555.2/μl), 15 of 79 patients had recurrences (19%), whereas 2 of 63 patients had recurrence in the group with a high lymphocyte count (>1555.2/μl) (3%) (*p* = 0.004). Univariate and multivariate analyses were performed to evaluate the relationships between clinical characteristics and patients’ outcomes. On univariate analyses, location (right colon vs left colon), tumor depth of invasion (T3 vs T4), CA19-9 (<37 vs ≥ 37 U/ml), lymphocyte count (≤ 1555.2/μl vs > 1555.2/μl), and PPLR (≤1.151 vs >1.151) were all associated with RFS (Table 4). Factors with *p* values < 0.05 on univariate analyses were included in the COX multivariate model analysis. On multivariate analysis, T4, a low lymphocyte count (≤ 1555.2/μl), and low PPLR (≤ 1.151) were independent predictors of poor RFS (Table 5).

**Table 4:** Univariate analysis for recurrence-free survival (RFS)

Parameter		RFS		
		p	HR	95% CI
Age (y)	< 60			
	≥ 60	0.51	0.56	0.10-3.12
Sex	Male			
	Female	0.14	0.4	0.12-1.36
Location	Right colon			
	Left colon	0.047	3.26	1.02-10.45
Differentiation	Well/Moderately/Pap			
	Poor/Muc	0.86	0.82	0.09-7.60
Tumor invasion depth	T3			
	T4	0.0053	6.71	1.76-25.56
Lymphatic involvement	Negative			
	Positive	0.32	2.41	0.42-13.85
Venous involvement	Negative			
	Positive	0.43	2.06	0.35-12.21
Diameter	< 5 cm			
	≥ 5 cm	0.31	0.52	0.15-1.84
CEA	< 5 ng/ml			
	≥ 5 ng/ml	0.91	1.07	0.34-3.35
CA19-9	< 37 U/ml			
	≥ 37 U/ml	0.075	3.67	0.88-15.34
Lymphocyte count	≤ 1555.2/μl			
	> 1555.2/μl	0.00016	0.03	0.00-0.18
PPLR	≤ 1.151			
	> 1.151	0.00044	0.11	0.03-0.38

PPLR: post/preoperative lymphocyte count ratio.

**Table 5:** Multivariate analysis for recurrence-free survival (RFS)

Parameter		RFS		
		p	HR	95% CI
Location	Right colon			
	Left colon	0.118	2.3	0.8-6.3
Tumor invasion depth	T3			
	T4	0.00003	10.1	3.4-29.9
Lymphocyte count (/μl)	≤ 1555.2			
	> 1555.2	0.0001	0.04	0.009-0.22
PPLR	≤ 1.151			
	> 1.151	0.0009	0.15	0.05-0.46

PPLR: post/preoperative lymphocyte count ratio.

### Scatter-plot of the preoperative lymphocyte count and the post/preoperative lymphocyte count ratio (PPLR)

**Figure 4:**
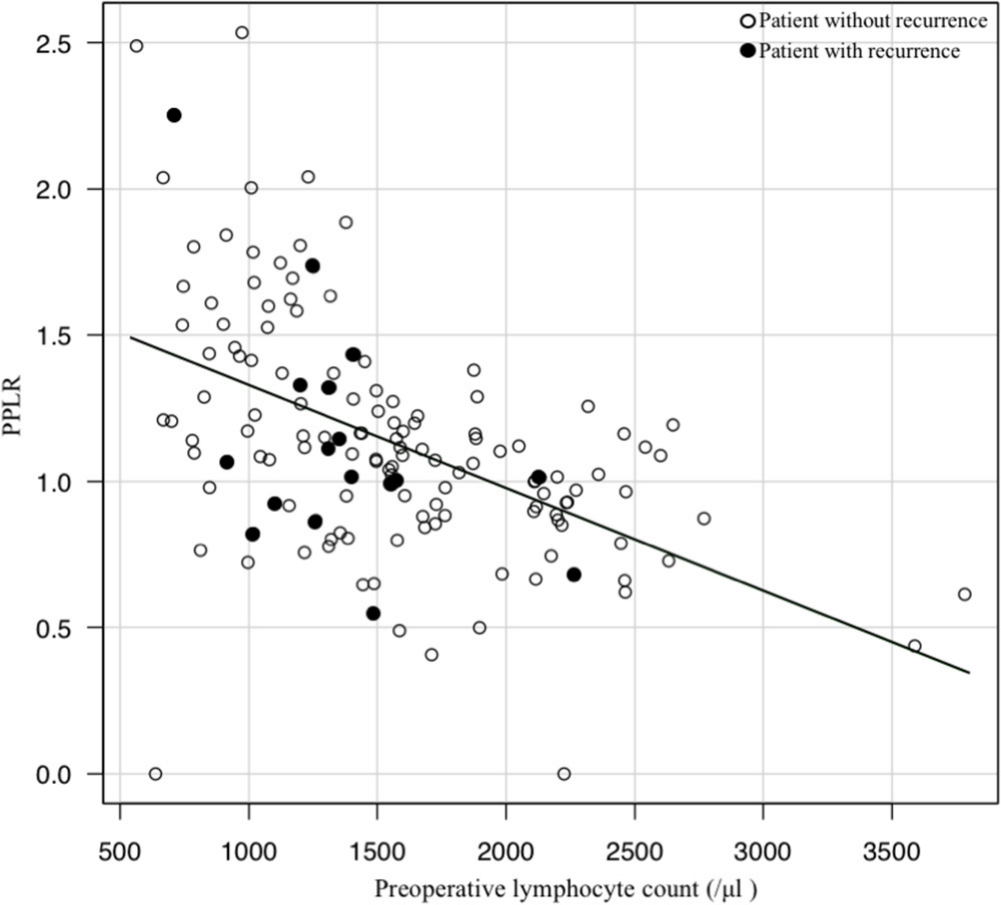
Scatter-plot of the preoperative lymphocyte count and the post/preoperative lymphocyte count ratio (PPLR) There is a significant negative correlation between the preoperative lymphocyte count and the PPLR on Pearson's product moment correlation coefficient analysis (*r* = −0.501, 95% CI: −0.615 −0.366, *p* < 0.01) The group with a low preoperative lymphocyte count and low PPLR has more frequent recurrences (white circle, patient without recurrence; black circle, patient with recurrence).

There was a significant negative correlation between the preoperative lymphocyte count and the PPLR (Pearson's product moment correlation coefficient, *r* = −0.501, 95% CI: −0.615 −0.366, *p* < 0.01). However, the group with a low preoperative lymphocyte count and low PPLR appeared to have more frequent recurrences (Figure 4).

### RFS and CSS curves categorized by the preoperative lymphocyte count and the PPLR

**Figure 5:**
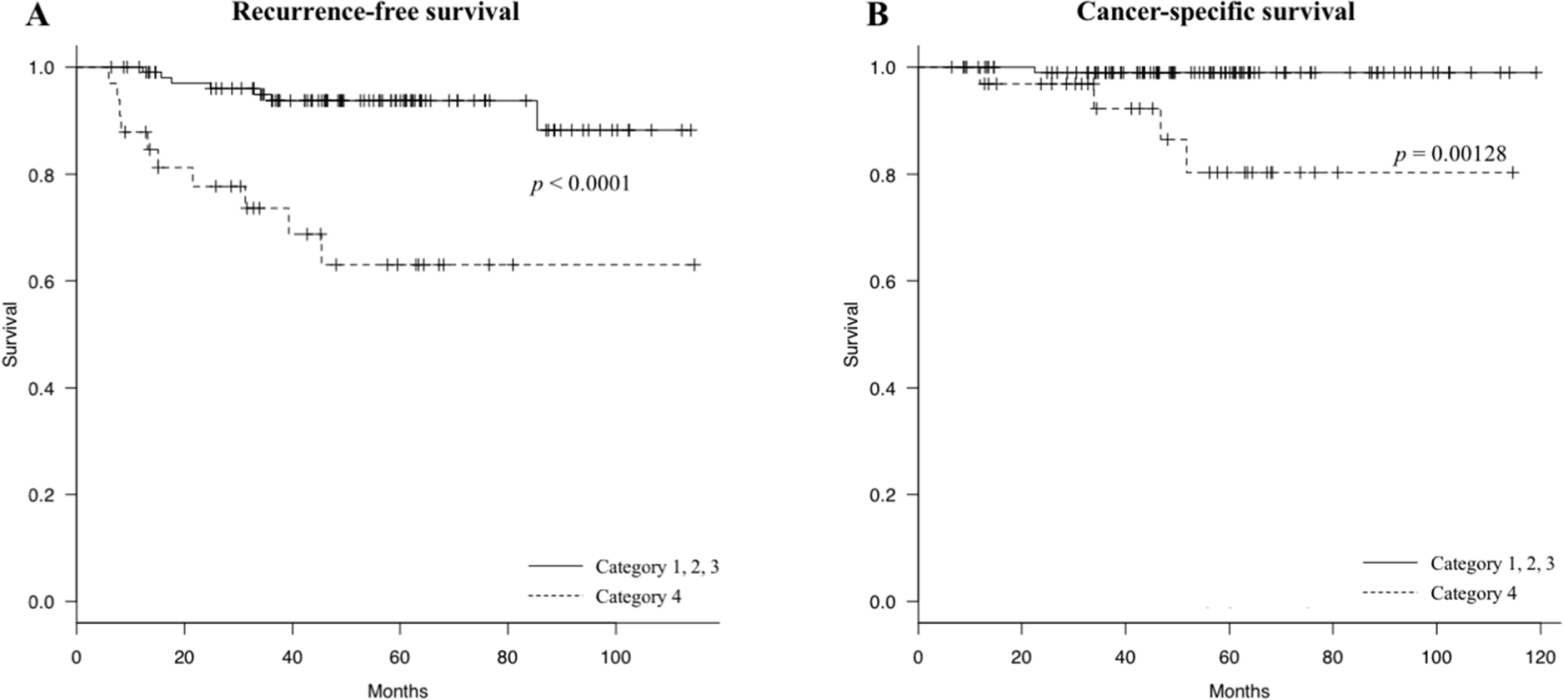
Recurrence-free survival (RFS) curves and cancer-specific survival (CSS) curves categorized by the preoperative lymphocyte count and the post/preoperative lymphocyte count ratio (PPLR) **(A)** Patients in category 4 (dotted line) show a worse RFS compared to the other categories (solid line) (log-rank *p* < 0.0001); **(B)** Patients in category 4 (dotted line) show a worse CSS compared to the other categories (solid line) (log-rank *p* = 0.00128).

Patients in category 4 had a worse RFS (log-rank *p* < 0.0001) (Figure 5A) and a worse CSS (log-rank *p* = 0.00128) (Figure 5B) compared to the patients in other three categories. These data suggest that the predictive accuracy for colon cancer recurrence based on lymphocyte number increases in combination with PPLR.

## DISCUSSION

CRC is one of the most frequent cancers affecting both sexes worldwide. Surgery and adjuvant chemotherapy with an oral uracil and tegafur plus leucovorin (UFT/UZEL) regimen have become standard therapy for patients with high-risk stage II and stage III colon carcinomas [3]. Nearly 25% of patients who undergo surgical resection for localized colon cancer will experience disease recurrence [10]. Although retrospective comprehensive studies showed strong associations between genetic mutations and the clinical outcomes of patients with CRC, no validated biomarkers are currently used in routine clinical settings [20]. Recently, components related to immunity have been of interest as diagnostic markers for CRC, including NLR [17] and PDW [17], and these diagnostic markers have a potential for practical use.

The association between colon cancer and lymphocyte counts has been examined in studies as early as the 1970s, in which a decrease in lymphocyte counts was found in patients with more advanced colon cancer [21]. Additionally, as one of the potential predictive markers, an elevated preoperative NLR was found to be a predictor of recurrence and worse survival after resection of CRC [17, 22, 23]. In the present study, RFS and OS of stage II colon cancer were found to be highly correlated with decreased lymphocyte counts, though preoperative NLR was not found to be an independent prognostic variable for recurrence of stage II colon cancer, as previously reported [23]. Furthermore, lymphocyte number and PPLR were combined, and it was possible to predict recurrence of colon cancer with higher accuracy than with lymphocyte number alone (RFS (*p* = 0.00238) and CSS (*p* = 0.0315) for lymphocyte number alone, RFS (*p* < 0.0001) and CSS (*p* = 0.00128) for lymphocyte number and PPLR together). These data suggest that patients whose preoperative lymphocyte number and PPLR are both low tend to have recurrence.

The lymphocyte count decrease in patients with recurrent CRC may be due to the possibility that patients who naturally have a low lymphocyte could more easily develop recurrent colon cancer. As to why PPLR is a predictive marker, it is considered that the lymphocyte counts increase after tumor resection (1548.0/μl before surgery, 1640.7/μl after surgery, *p* = 0.046; data not shown). These data indicate that lymphocytes are accumulated and consumed at the tumor and metastatic foci, as shown in previous reports [24, 25], and/or that tumors inhibit lymphocyte production ability in the bone marrow, presumably through soluble factors including exosomes [26, 27]. Therefore, after successful cancer resection, postoperative lymphocyte counts should increase compared to preoperative lymphocyte counts. Thus, a low PPLR indicates that there could be still remaining tumors or micrometastatic foci.

For patients with a low lymphocyte count and a low PPLR, a better drug treatment would be one that would increase the number of lymphocytes, such as protein-bound polysaccharide kureha (Kureha Corporation, Tokyo, Japan), Z-100 (ZERIA Pharmaceutical Corporation, Tokyo, Japan) [28, 29]. Furthermore, immune-enhancing nutritional supplements may be effective [30, 31]. The advantage of this approach is that lymphocyte counts and PPLR are easy and inexpensive to monitor. In addition, identifying the types of lymphocytes that are reduced would be informative for the development of new drugs and therapeutic approaches. In order to clarify this, further studies using flow cytometry and antibodies for lymphocytes such as T and B cells would be needed. In sum, the number of lymphocytes and PPLR could be used in clinical settings to predict the prognosis of patients with colon cancer after curative resection.

In conclusion, the combination of the lymphocyte count and the PPLR appears to be a potential marker for predicting recurrence of stage II colon cancer. Further study is needed to determine whether lymphocyte counts have a direct correlation to recurrence of stage III colon cancer. Since stage IV colon cancer already has metastases to other organs, a study examining the relationship between overall survival/progression-free survival during chemotherapy and the combination of the lymphocyte count and the PPLR would be needed. Additionally, the types of lymphocytes that are reduced also requires further clarification.

## MATERIALS AND METHODS

### Patients and clinical follow-up

A retrospective review of a database with 142 patients who had undergone curative surgery for histological TNM stage II colon cancer between January 2008 and December 2014 at a single institution (Nippon Medical School Hospital, Bunkyo-ku, Tokyo, Japan) was conducted. Disease stage was established in accordance with the AJCC 7th classification. The exclusion criteria included: emergency surgery (colorectal cancer with intestinal perforation or obstruction), 10 patients; double cancer, 39 patients; period of observation less than 180 days, 18 patients; and incomplete clinicopathological data, 21 patients (Figure 1). Fourteen of 142 patients received adjuvant chemotherapy after surgery, including oral adjuvant uracil and tegafur plus leucovorin (UFT/UZEL) in 11 patients and capecitabine in 3 patients. Follow-up investigations included clinical check-ups, laboratory measurements (including routine blood examinations and cancer-related marker analysis, such as CEA and CA19-9, every 3–6 months), radiological assessment (abdomen and chest computed tomography, every 6–12 months), and colonoscopy (one and three years after the surgery). All patients were followed-up from 6.1 to 116.3 months after surgical treatment. RFS was defined as the interval from radical surgery to recurrence. This study was conducted in accordance with the principles embodied in the Declaration of Helsinki and approved by the ethics committee of Nippon Medical School. (Registration no. 29-07-781).

### Clinicopathological data

All patient-related data were retrieved from the medical record database, including blood test values, some biochemical indicators such as serum albumin levels and serum CEA and CA19-9 levels, as well as demographic information and postoperative pathological results. Preoperative blood laboratory tests were carried out within 30 days before surgery, and postoperative blood laboratory tests were performed between 21 and 90 days after surgery. NLR was defined as the serum absolute neutrophil count divided by the serum absolute lymphocyte count, and PPLR was defined as the postoperative serum absolute lymphocyte count divided by the preoperative lymphocyte count in peripheral blood. NLR and PPLR were calculated for each patient.

### Combined use of the preoperative lymphocyte count and the post/preoperative lymphocyte count ratio (PPLR)

The patients were divided into four categories based on their preoperative lymphocyte count and PPLR. Patients with a high preoperative lymphocyte count (>1555.2/μl) and high PPLR (>1.151) were categorized into category 1 (n = 11). Patients with a high preoperative lymphocyte count and a low PPLR (>1555.2/μl, ≤1.151) were categorized into category 2 (n = 52). Patients with a low preoperative lymphocyte count and a high PPLR (≤1555.2/μl, >1.151) were categorized into category 3 (n = 46). Patients with a low preoperative lymphocyte count and a low PPLR (≤1555.2/μl, ≤1.151) were categorized into category 4 (n = 33).

### Statistical analysis

The chi-squared test or Fisher's exact test were used for categorical variables. Quantitative data are presented as means ± standard deviation and compared using the Mann-Whitney U test. Pearson's test was used for correlations. Univariate analysis was performed to evaluate clinical characteristics related to RFS. On multivariate Cox regression analysis, the model was adjusted for prognostic clinicopathological factors on univariate analysis. Hazard ratios estimated from the Cox regression analysis are reported as relative risks with corresponding 95% confidence intervals. Survival curves were prepared using the Kaplan-Meier method and compared by the log-rank test. All statistical analyses were performed with EZR (Saitama Medical Center, Jichi Medical University, Saitama Japan), which is a graphical user interface for R (The R Foundation for Statistical Computing, Vienna, Austria). More precisely, it is a modified version of R commander designed to add statistical functions frequently used in biostatistics [32]. All analyses were two-sided, and a *p* value of <0.05 was considered significant.

## Abbreviations

ROC: receiver operating characteristic
PPLR: post/preoperative lymphocyte count ratio
CA19-9: carbohydrate antigen 19-9
RFS: recurrence-free survival
CSS: cancer-specific survival
CRC: colorectal cancer
CEA: carcinoembryonic antigen
SIS: systemic inflammation score
LMR: lymphocyte and monocyte ratio
NLR: neutrophil and lymphocyte ratio
PLR: platelet to lymphocyte ratio
PDW: platelet distribution width
EGTM: European Group on Tumor Markers
AUC: area under the curve
CI: confidence interva
